# The efficacy of the corpus-based error correction method on revision in writing classrooms

**DOI:** 10.1371/journal.pone.0317574

**Published:** 2025-03-12

**Authors:** Yiyang Yang, Hulin Ren

**Affiliations:** School of Foreign Studies, University of Science and Technology Beijing, Beijing, China; Educational Testing Service: ETS, UNITED STATES OF AMERICA

## Abstract

Despite the growing interests in investigating the application of data-driven learning (DDL), much existing research remains outcome-oriented. Limited attention has been paid to learners’ interactions with corpora, especially the experiences of consulting corpora and decision-making processes during revision in second language (L2) writing. In this regard, this study investigates how corpora assist language learning during the revision process in a classroom-based foreign language learning context. We recruited 123 non-English major undergraduates from a university in China, learners’ revision behaviours and outcomes were analysed in each draft. To gather the learners’ experiences and perceptions of using the BAWE corpus for error correction, a stimulated recall interview was conducted using the revised drafts as stimuli. Quantitative results indicated that corpus consultation was highly effective for revising word form errors (93.6%), and participants were more likely to use the BAWE corpus to correct collocation and phrase errors (62.7%). Interview results demonstrated that the corpus-based error correction method was effective in enhancing correctness and facilitating language learning. Learners were able to utilise corpora to identify patterns, shape writing habits, and test linguistic hypothesis. The findings and the implications of this study also provide valuable insights for teachers into the potential role and implementation of corpora in designing classroom writing tasks.

## Introduction

As a collection of texts or speech transcriptions, corpora have been used by academic researchers in the fields of computational linguistics, lexicography, writing, and other linguistic areas for over half a century [1]. From a pedagogical perspective, corpora can be used indirectly through a paper-based approach where teaching materials are developed from corpus output to offer learners more authentic examples, or directly through a hands-on approach, allowing learners to seek language patterns directly through corpus query [2]. The implementation of either approach can be categorised into data-driven learning (DDL). DDL offers language learners a great amount of language input (i.e., through corpus query results or teaching materials), enabling them to learn language patterns and solve language issues without the need to consult more proficient language users [3]. In this way, students can receive language input from corpora, and develop their independence to detect and apply language patterns by themselves inductively, as well as to gain linguistic knowledge deductively with support from teachers [3–6]. Although DDL has still not been extensively implemented in English learning contexts because of the affordance of corpora in remote areas [7], it has gained considerable attention from educators and researchers. By implementing direct DDL, this study aims to discover how consulting corpora could enhance English writing during the revision stage.

## Literature review

### DDL and error correction

DDL research has undergone a period of rapid growth since 2005 [8]. The pedagogical functions of DDL in language classrooms include providing authentic examples, facilitating writing and/or speech processes, developing learning skills, and bringing growing enjoyment and engagement to learners [9–11]. By making corpus queries, information on the frequency of expressions drawn from authentic examples of English (i.e., expressions written by British undergraduates from the British Academic Written English Corpus) can be obtained by corpora users, immersing learners within an authentic language environment.

Research has demonstrated the effectiveness of DDL in enhancing vocabulary acquisition and written production. Chan and Liou [12] observed significant improvement among Taiwanese L2 learners in acquiring collocation knowledge through web-based consultations using a bilingual corpus tool. Frankenberg-Garcia [13,14] found that the corpus output helped L2 learners understand target vocabulary, correct typical errors, and use vocabulary more accurately in sentence construction and written production. Similarly, Gilmore [15] reported more natural language production in learners’ subsequent writing drafts after implementing corpus-based error correction methods. The concordance, by providing sufficient and authentic language data, thus supports learners in making appropriate language choices [16]. Among the related studies, one of the common ways of employing DDL in L2 writing classrooms is to associate the use of corpora with written corrective feedback (WCF).

Available evidence shows that language learners can benefit from DDL when using corpus-based correction methods as part of WCF. WCF refers to the feedback on the linguistic problems in learners’ written texts. It is offered by teachers or peers as a way to help language learners improve accuracy in their writing and facilitate language acquisition through the revision process. According to Ellis [17], WCF types include direct and indirect CF, metalinguistic CF, electronic feedback, and reformulation, as illustrated in Table 1 below.

**Table 1:** Types and the scope of corrective feedback^a^^.^

Classification of CF	Description
**Direct CF/ Reformulation**	Teacher provides the student with the correct form.
**Indirect CF**	
1. Indicating and locating	Teacher underlines or highlights errors.
2. Metalinguistic CF	Teacher writes codes in the margin (e.g., WC = word choice; art = article) or marks errors with grammar descriptions.
**Electronic feedback**	Teacher indicates the error with ^a^ hyperlink to concordance.

^a^Adapted from [17].

WCF types may affect the time and effort required in revision preparation and processes for both teachers and learners. Direct WCF and reformulation refer to teachers providing correct answers to errors, while indirect WCF means teachers locating the errors without offering corrections. The provision of direct WCF varies as the corpus-based direct WCF offered to learners is to identify and/or locate errors by showing them pre-selected corpora outputs, whereas the traditional direct WCF provision like recast provides accurate expressions to learners directly.

The distinction between the corpus-based direct feedback type and the traditional direct WCF proposed by Ellis is that, learners need to identify appropriate language patterns (that is, the ‘correct form’) from the pre-selected data when receiving corpus-based direct WCF, which is similar to the provision of electronic feedback that has been identified by Ellis. The implementation of corpus-based direct WCF involves locating errors and providing pre-cast hyperlinks to relevant corpus results [18–20], requiring language teachers to initiate corpus consultation. For instance, Gaskell and Cobb [19] incorporated URL links of online concordance results directly into texts alongside each error using track changes, while in Boulton & Landure [20], each error was identified with feedback using MS Word comments, with providing links to COCA (the Corpus of Contemporary American English) concordance results. Boulton & Landure [20] focused on demonstrating methods for corpus-based feedback, thus limited discussions regarding the usefulness of DDL were provided. In contrast, Gaskell and Cobb [19] found corpus-based direct WCF effective in promoting learner autonomy, leading to independent corpus consultation.

On the other hand, the corpus-based indirect WCF, unlike the direct one, requires learners to actively engage in corpus consultation by making their own corpus queries before integrating and applying corpus output. Offering indirect feedback such as highlighting and coding [21–24] have shown effectiveness in reducing errors [25–27]. Associating with corpus-based indirect WCF, Corpus linguistics and L2 writing researchers have attempted to investigate learners’ use of corpora during the revision stage, examining how and to what extent learners can correct marked errors by corpus concordancing. Liou [21] provided learners with an error list for revision at home, while Crosthwaite [22] underlined errors in learners’ written drafts without any additional commentary. Bridle [23] and Dolgova & Mueller [24] coded the errors with error types indicated. Regardless of the specific approach to offer corpus-based indirect WCF, these studies found the corpus-based indirect WCF useful for resolving issues with collocation, lexicogrammar, omissions, and additions. To promote independent corpus consultation, the corpus-based indirect WCF will be used in this study, as it is more learner-centred.

As a reference tool to facilitate L2 writing, different types of corpora serve distinct roles. Particularly, the British Academic Written English (BAWE) corpus supports those aiming to analyse academic linguistic patterns, while Sketch Engine for Language Learning (SKELL) corpus is tailored for general language learners. Broader resources like the British National Corpus (BNC) and the Corpus of Contemporary American English (COCA), collect a diverse range of written and spoken materials from media for varied ages and public interests. In L2 writing research, Gilmore [15], Bridle [23] and Şahin Kızıl [28] employed freely available BNC, whereas researchers like Liou [21], Dolgova & Mueller [24], and Satake [26] used COCA with their participants. With providing corpus training lasting from 20 to 90 minutes, these studies prepared their participants for making corpus queries independently to address various language issues. Similarly, Crosthwaite [22] implemented a 2.5-hour corpus training program for learners, educating them to consult SKELL and BNC via Sketch Engine platform for all types of language problems. Crosthwaite [29] further designed the training program with the use of the BAWE corpus, and found it effective in revising lexical errors especially. The training duration ranges from under two hours to over ten sessions, with consistent positive effects observed in pre/post-test designs across all durations [30].

Regarding number of errors coded in studies on the efficiency of the corpus-based WCF, most researchers (e.g., [15,21,24–26]) opted to code and record all errors regardless of error types. While Satake [26] required learners to revise at least one error with consulting the corpus, other studies allowed learners to choose their preferred correction tools, with reporting the percentage of errors revised by corpus consultation. Generally, about ten errors per essay were corrected in these studies. Cheng [25] coded 14 types of language errors, marking 10-15 errors per essay. Similar to [15], percentages on the use of reference tools were reported. In contrast, [19] and [27] coded five and two errors per essay, respectively, requiring all errors to be corrected using corpora. These studies covered a comprehensive range of error types to compare the effectiveness of corpora consultation on grammatical/lexical or local/global error distinctions. However, investigations into the specific effectiveness of corpus consultation for a more focused error types remains underdeveloped.

Another confounding factor in WCF is the type of errors coded, and related literature has demonstrated the impact of DDL on various error types. Literature shows that learners are more successful in revising collocation [22,31,32], word choice [22,25,32], and omission and addition problems [27,33], compared to resolving preposition misuse [27] and grammatical issues [22,34]. By comparing the rate of successful corrections and/or the number of erroneous items produced with corpus consultation to those produced without corpus consultation, empirical evidence reveals the effectiveness of the corpus use on correcting collocation and phrase errors, particularly on omission in verb phrases [26,33]. Through essay writing and revising tasks, both [26] and [27] found omission and lexical errors were highly successfully resolved with corpus consultation, as compared to correcting other types of errors with learners’ own knowledge or dictionaries. Participants in [25] relied more on corpora for correcting word form errors, followed by word choice errors. Similarly, Liou [21] pointed out that learners preferred using corpora for correcting verb formation issues, followed by word form, word choice and preposition errors. However, participants were more likely to use their own knowledge for punctuation and grammatical corrections, such as subject-verb agreement, comma splice and verb tense. Through three revision tasks, the error rate in the final writing task in [21] decreased by 70%, with improvement especially in verb phrase, verb tense and preposition issues, as demonstrated by two case studies. Findings in [7] also emphasise that corpus consultation is particularly useful for revising lexical errors, reinforcing their previous findings in [22] that this is more effective in revising word form, word choice, collocations and phrases rather than morphosyntactic and deletion issues with corpus consultation. Therefore, DDL proves more valuable for dealing with vocabulary and phrase issues than grammatical issues.

It is thus relatively less useful and more time-consuming to address grammatical issues by consulting corpora because grammatical rules can be explained clearly and explicitly without the process of pattern finding. In contrast, the use of lexical items requires learners to understand the context of using such features, which is more difficult to explain in words. Such recent findings suggest that corpus-based error correction methods may bring relatively more successful error resolution on lexical problems. Therefore, to contribute to the literature in exploring the efficacy of corpus consultation for error correction with a more focused error types, this study implemented a focused and indirect corpus-based WCF approach, with focusing on three types of lexical errors rather than all types of errors.

### DDL and learners’ proficiency

Previous studies, especially review articles, have considered learners’ proficiency as a factor in DDL practices for L2 writing. Most research has recruited intermediate learners, aligning with the Common European Framework of Reference for language (CEFR) level B1 to A1 (e.g., [21,24,26,28]). As reviewed in [35–37], more than half of DDL research in L2 writing has been condcted at the university level, focusing on intermediate to upper intermediate level undergraduates. Interestingly, DDL has been found more effective among lower-level learners (e.g., [38,39]), and Boulton & Vyatkina [37] advocates for more works targeting on learners with lower levels of proficiency regardless of age. Therefore, this study considers participants with an overall proficiency below B1 to address the call for investigating the effectiveness of DDL at lower proficiency levels.

### The efficacy of DDL and learners’ perceptions

Most findings from the above-mentioned literature are drawn from quantitative analysis of revision outcomes (also see [40]), but the efficacy of corpus consultation has not been sufficiently revealed qualitatively through learner-corpus interactions (see [35]). Many researchers have attempted to collect retrospective data (e.g., questionnaires, interviews, journals) as supporting evidence to reveal the efficiency of the corpus-based error correction method. In general, alongside with positive evidence demonstrated by numerical data, learners’ perceptions towards the use of corpora were found positive: they perceived it as a useful reference tool in enhancing English writing development, in terms of the accessibility of multiple corpora on a single platform and vocabulary acquisition [25,33,41–43]. However, Reynolds [31] showed the opposite regarding learners’ attitudes towards corpus use. In self-correcting collocation issues in academic writing, his participants expressed negative attitudes as they perceived correction work as the teacher’s job, and found consulting the corpus unnecessarily difficult. Furthermore, challenges of learners’ use of corpora have been noted in the studies that received generally positive attitudes from participants towards the use of the corpus-based error correction method. These challenges include difficulty in working with corpus interfaces, lack of confidence in applying the detected patterns, and the process being time-consuming and laborious (e.g., [31,41,44–47]). Although corpus-based error correction methods are found effective in improving revision outcomes, those previous studies show that how learners’ experiences in using the methods on particular types of error needs further investigation.

While some previous works have revealed learners’ perceptions towards the corpus-based error correction to a certain extent, the thinking processes they engage in during corrections, and their interactions with the corpus remain underexplored. Given the limited insight into how leaners reach these perceptions and navigate the corpus consultation processes when making corrections, this study aims to address this gap by exploring learners’ experiences and decision-making processes during corpus-based revision in L2 writing.

To sum up, the majority of the previous studies, are still product-oriented, with some using the retrospective data as supportive evidence to evaluate the effectiveness of DDL. Thus, there is still limited understanding of the interactions between learners and corpora, especially during the revision processes in research regarding L2 writing. Therefore, by focusing on a limited range of error types and implementing a mixed-method approach with learner-corpus interactions dominating the investigation, this study attempts to answer the following two questions:

RQ1: To what extent can a group of Chinese learners of English revise lexical errors successfully by using a corpus?
RQ2: What are these learners’ thoughts and experiences of the use of the corpus-based error correction method?

## Materials and methods

### Participants and settings

Convenience sampling was used to select a university in Southwest China (hereafter referred to as SW University) as the research site based on availability and time. The participants in SW University were purposively recruited according to their linguistic characteristics (i.e., Chinese English learners, proficiency) and institutional characteristics (i.e., non-English major, enrolled in the same writing course). More specifically, the selected participants were majoring in Computer Science or Engineering, with below B1 level of English writing proficiency according to CEFR. This proficiency level was determined based on their recent College English Test Band 4 (CET 4) scores (the completion of CET4 mostly corresponds to the CEFR B1 level [47], while a CET 4 score of 425-450 aligns with the CEFR B2 level [48]). The average age of the participants is 19. None of them had previous experience or training in using corpora for English writing. Initially, 131 undergraduates were recruited; however, since eight of them did not attend the following research activities, their data was removed. In total, the data from 123 students was analysed. All participants were informed of the purpose and procedure of the research and gave consent. All identifiers were removed before data analysis. Table 2 presents the background information of the participants.

**Table 2:** Demographic and background information of participants.

Background Survey Components	n	%
**Major**		
Computer science	68	51.9%
Engineering	59	45.1%
Not specified	4	3%
**English learning time per day**		
~30 mins	41	31.3%
~60 mins	59	45.0%
2-3 hours	31	23.7%
**Self-reported proficiency level**		
Below B1 level	106	80.9%
B1 level	21	16.0%
B1-B2 level	4	3.1%

The course that the participants enrolled in was an entrance level writing course (the pseudonymous course code EWRT202) held for physical science undergraduates at SW University. Activities in EWRT202 focus on developing the learners’ logical thinking and writing, as an initial step towards teaching the students to produce acceptable abstracts in English after completing the one-year academic writing course series.

The written pieces were produced and collected in pre-arranged writing tests based on the teaching schedule, which served the purpose of formative writing practice with the aim of progress tracking. The writing topics were chosen by the classroom teacher according to the teaching plan. The argumentative essays were produced under the same conditions as the final writing examination of the course, that is, to write a 150-word argumentative essay under timed and invigilated conditions. The implementation of the research aligned with the teaching activity, which means that the production of texts was part of the learning activities.

### The corpus

The corpus used in this study was the British Academic Written English (BAWE) Corpus, a freely available reference for language learners that offers access to English word collocations, phrases, and thesauruses. The BAWE Corpus is freely accessible online via the Sketch Engine as well as Lextutor, and it was employed in this study via Sketch Engine (See: https://app.sketchengine.eu/#dashboard?corpname=preloaded%2Fbawe2). The BAWE Corpus was initiated and created at the University of Warwick, comprising high-standard students’ assignments from three UK universities, encompassing approximately 3,000 assignments and 6.5 million words [49]. These assignments span four main disciplinary areas: arts and humanities, social science, life science, and physical science [50]. As an expert corpus that is widely used in corpus linguistic studies to compare with other learner corpora, BAWE corpus provides standard language samples for English learners at the university level (e.g., [51,52]). Given that the participants in this study were from computer science and engineering disciplines, the physical science discipline of the BAWE corpus was particularly relevant.

### Error taxonomy

After the essays were collected, we analysed them for error types. The coding scheme of error types used in this study was adapted from James [53]. James suggests five major error categories for surface structure: omission (deleting necessary components), addition (adding unsuitable items, excluding blends), misformation (using incorrect forms), misselection (misunderstanding meaning and usage), misordering (incorrect word order), and blends (combining a part of two related structures when either of which serves the purpose). James further defines four types of lexical errors, namely, misselection (wrong choice of words that look/sound/mean similar), misformations (creating non-existent words/phrases), distortions (including omission, over-inclusion, misselection, misspelling and blends at the lexical and phrase level), and collocations.

This study focused on lexical errors, among which collocation [22,31,32], word form [22], word choice [22,25,54], and omission and addition problems [27,33] were found in the literature to be more effectively revised by consulting corpora. Consequently, other error types identified by James under the lexical category, for example, over-inclusion, misspelling and misordering (i.e., wrong letter sequence) were unmarked. Aligning with the error types focused on in this study, the following categories were retained and renamed:

Word form (WF) issues: related to misformation (using inaccurate forms);

Word choice (WC) issues: related to misselection and misformation (creating non-existent words) of a word, a whole phrase, or the verb in a verb phrase;

Collocation and phrase (C/P) issues: related to omission, addition or misselection of a preposition in verb phrases, incorrect word choices within noun phrases, and blends of two similar phrases.

After coding three sample essays at the beginning of the feedback provision period, two raters, who were the first author and the tutor of EWRT202, carried out a discussion of initial differences in the proposed coding. Further, to reduce any confusion arising from discrepancies between the coded errors and the output presented by the BAWE corpus, the corpus query output was carefully reviewed at this stage. Based on this review, an agreement was reached between the two raters on lexical error taxonomy and criteria. Other error types related to clauses (i.e., linking words of clauses), syntax (i.e., word order), grammar (i.e., tenses, subject-verb agreement), spelling, articles and global errors were excluded and not coded. Table 3 presents the adapted error taxonomy with examples. Participants were instructed to revise only the coded errors. While they were not prohibited from correcting unmarked errors, data for such corrections were not collected for analysis.

**Table 3:** The categorisation of the three types of focused error coding.

Types	Description	Examples
**WF**	**Misformation**: The use of the wrong form of a word (excluding tense and singular/plural issues)	E-learning bring lots of *convenient*.
**WC**	**Misselection/misformation**: Inappropriate choice/creation of a a word. b the verb of a verb phrase. c a phrase/collocation as a whole	But, this change also changes people’s *means* about traditional study.
**C/P**	**Omission/ addition/ misselection within phrases** (but not the whole phrase is erroneous): a Within a noun phrase. b Prepositions in verb phrases. **Blends** (combining two similar collocations).	The second one is that we have numerous ways to study and not just limited to *listen teachers* on class. This question is *easy to be answered* * *combining easy to answer and easily answered

### Ethics

While the raw data of the study was obtained during the first author’s MA program, the human ethics protocol (GU reference no. 2021/792) received full approval from Griffith University, where the first author completed her MA study. The recruitment period spanned from October 26, 2021, to December 30, 2021. All participants gave verbal and behavioural consent, with verbal consent obtained during the interview process. Participants were informed that their involvement in the study was voluntary, and they could withdraw from the study at any point.

## Error corrections in written drafts

### Data collection procedure

**Fig 1:**
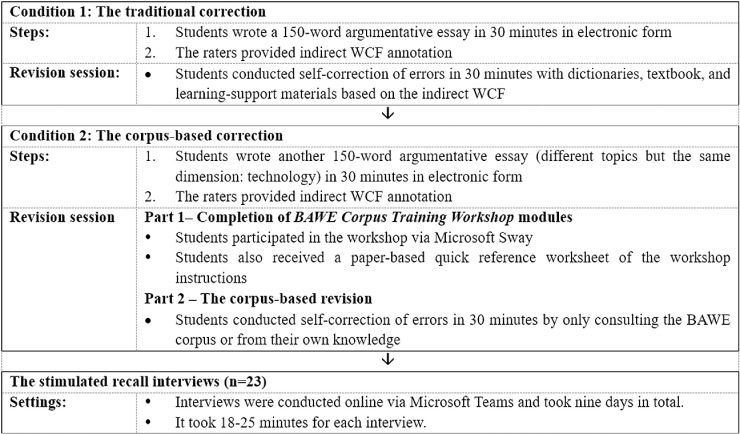
Overview of data collection procedure timeline.

The present work is a part of a larger study regarding the investigation of the efficacy of the corpus-based error correction method in tertiary language classrooms in China. In the course of the study, we collected learners’ written drafts across five weeks, as demonstrated in Fig 1. Indirect WCF were provided in Draft 1 and Draft 2 on three types of lexical errors, namely, word form (WF), word choice (WC), and collocation and phrases (C/P) errors, by coding them using the ‘Comment’ function of Microsoft Word with error types identified.

In the first two weeks, students wrote a 150-word argumentative essay within 30 minutes in electronic form. A week following this, the traditional error correction session began, and the participants were allowed to use electronic dictionaries and supportive materials such as teaching slides and study notes to resolve the coded errors in thirty minutes. They were also informed that they could leave the errors that they do not know how to correct unrevised. The data collected from this draft were not discussed in this paper since it is irrelevant to the research foci, the purpose was to familiarise students with the error codes and error correction format.

**Fig 2:**
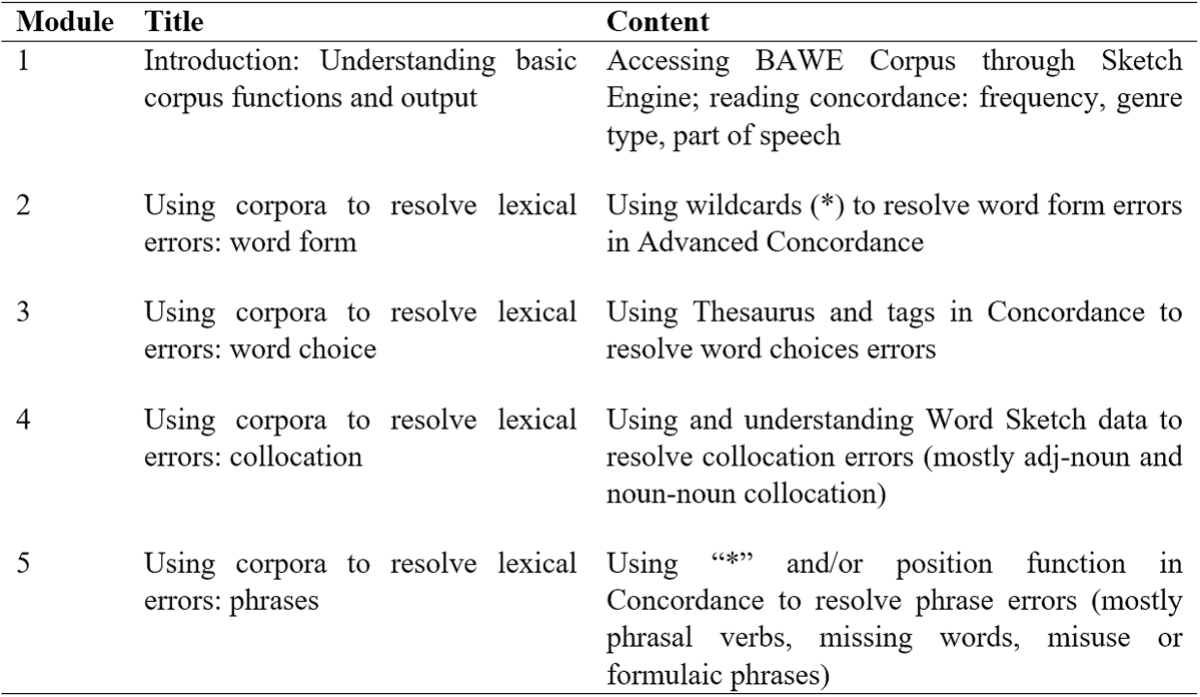
Overview of the content of the workshop modules (adapted from [29]).

After familiarising students with the error codes through the previous revision session, the second condition commenced in the final three weeks. Students were asked to write another 150-word argumentative essay on a different topic within the same category, technology, in 30 minutes. With the same types of error coded, participants were instructed to correct the coded errors in the following week. On the same day but prior to the corpus-based revision session, participants attended a computer-based training session entitled the BAWE Corpus Workshop in class. The training module, which lasted for 50 minutes, was adapted from Crosthwaite [29] and created by the first author in the form of instructional texts and videos in Chinese. The modules aimed to equip participants with the skills to consult the BAWE Corpus via Sketch Engine for self-correction of the coded items. Another thirty-minute practice session was also held as part of the workshop, allowing learners to practice consulting the BAWE corpus to resolve pre-prepared errors and clarify any doubts regarding the use of corpora. Fig 2 briefly illustrates the content of the training module.

Ten minutes after the workshop, a thirty-minute corpus-based revision session began. In addition to the online instructions, participants were provided with a paper-based handout that included a quick reference of resolving different lexical issues. They could consult either the online workshop modules and/or the handout during the corpus-based revision session. Dictionaries and other support materials were prohibited to encourage learners to use the BAWE corpus as the reference tool. However, to follow a natural revision behaviour typically observed in language classrooms, participants could also revise the coded errors using their own knowledge, making corrections without relying on any reference tools. As long as the BAWE corpus was the only reference tool consulted, their revision behaviour was not restricted. Participants were required to document their revision decisions next to each error correction. The numerical data collected from learners’ corrected drafts were analysed to address RQ1, demonstrating the extent to which learners benefited from the corpus-based error correction according to the revision outcomes.

### Data analysis methods

The number of corrections and successful corrections in learners’ drafts were collected. The rate of correction and the rate of successful correction were calculated using the following equations:


$$\text{Rate of correction}=\frac{\text{Number of corrections}}{\text{Number of coded errors}}\times 100\text{\%}$$

$$\text{Rate of successful correction}=\frac{\text{Number of successfully corrected errors}}{\text{Number of corrections}}\times 100\text{\%}$$

$$\text{Rate of corpus based successful correction}=\frac{Numberofcorpusbasedsuccessfulcorrections}{Numberofcorrectionsrevisedwithcorpus}\times 100\%$$

A Chi-squared test and a logistic regression test were then implemented to analyse the relationship between error type, the frequency of successful corrections and learners’ decision on the use of corpus.

### Results: Error type distribution

**Fig 3:**
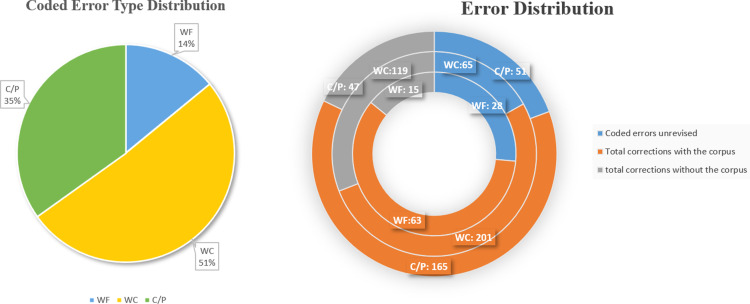
Error and Correction distribution.

To answer RQ1, we noted the number of errors corrected by the participants and their choices of using or not using the corpus for making corrections, Table 4 shows the number of errors corrected with and without the BAWE corpus, the distribution of error corrections as well as the number of successful corrections of each choice. With all errors across the three types coded, participants made an average of 6.1 errors per essay. Fig 3 presents the error correction distributions. On average, each participant made one error in WF, three errors in WC, and two errors in C/P. To summarise, WC errors comprised a relatively large proportion of the total number of errors, followed by C/P and then WF errors, which indicates that word choice issues are the most prevalent lexical problem among this group of Chinese learners.

**Table 4:** Distribution of error correcting in the corpus-based revision session.

Error type	Total errors	Coded errors unrevised	Corrections with the corpus		Corrections without the corpus	
**Successful**	**Total**	**Successful**	**Total**
**WF**	106	28	59 (93.6%)	63 (59.4%)	12	15
**WC**	385	65	116 (57.7%)	201 (52.2%)	77	119
**C/P**	263	51	104 (63.0%)	165 (62.7%)	34	47
**Sum**	**754**	**144**	**279 (65.0%)**	**429 (56.9%)**	**123**	**181**

*Note.* WF refers to word form errors, WC to word choice, C/P to collocation and phrase. “Corrections with the corpus” denotes the number/rate of corrections that learners made based on corpus results during the corpus-based revision session, while “Corrections without the corpus” refers to the number/rate of corrections that learners chose to correct by themselves without referencing any external tools during the corpus-based revision session.

### Results: Error correction distribution

More than half of the errors of each type were revised by consulting the corpus (Fig 3). In particular, participants attempted to correct C/P errors with the use of corpus more. A logistic regression analysis was run on the dataset of 754 errors with the error type as the independent variable and the corpus use (with/without) as the dependent variable. Table 5 presents the logistic regression results with a predictive power (χ^2^ =  122.587, *p* < .001). In general, learners tended to revise the three types of errors using the corpus. The results further suggest that the participants were more likely to revise C/P errors (*z* =  7.595, Wald =  57.688, *p* < .001) by consulting the corpus, followed by WC errors (*z* =  4.532, Wald =  20.538, *p* < .001) and WF errors (*z* =  4.995, Wald =  24.951, *p* < .001).

**Table 5:** Logistic regression model results (with and without using the corpus).

				Wald Test	
Error type	Estimate	SE	*z*	Wald	*p*
C/P	1.256	0.165	7.595	57.688	<.001
WC	0.524	0.116	4.532	20.538	<.001
WF	1.435	0.287	4.995	24.951	<.001

This finding is similar to Crosthwaite [22], in which the participants had various L1 backgrounds. His study found that participants were more likely to use corpora to correct phrase and collocation errors, followed by correcting word choice errors and then word form errors. The present findings also partially align with those in Satake [26], where, among 24 types of errors, learners chose to revise errors of omission (including omitting words of collocation, which was categorised as a C/P error in this study), addition, collocation and word order more by consulting corpora; as well as in Bridle [23], whose participants preferred using corpora to revise “Wrong Word” issues.

### Results: Revision accuracy distribution.

A Chi-squared test result suggested a significant difference in correction accuracy of the three error types (χ^2^ =  26.420, df =  2, *p* < .001, V = .208). Further analysis showed that a significant difference by the corpus use was found in accurate and inaccurate revision of WF errors only (*p* < .05). These results show a moderate association between learners’ correction accuracy and error type, and an interaction between the choice of correction method and accuracy in revising WF errors, which may imply referring to the corpus results is more effective in revising WF errors. The results slightly differ from previous studies. Literature shows that corpus consultation has been shown to be effective on all three types of error [22,25,31,54], but the present results only suggest its effectiveness on successful correction of WF errors.

## The stimulated-recall interview

### Data collection procedure

The invitation of online stimulated recall interview was sent to all participants after finishing the final revision session. Twenty-three students accepted the invitation and volunteered to participate. The stimulated recall interviews were conducted online via Microsoft Teams and recorded. They started three days after the revision session, and spanned nine days. The stimulated-recall interviews collected data pertaining to participants’ perceptions, behaviours, and thinking processes when making corrections, using participants’ revised essays as stimuli. Participants were also encouraged to demonstrate how they complete corpus queries during the interviews. Those data were collected to resolve RQ2. The interview data could reveal learners’ thoughts and opinions towards the use of corpora, which reflects learners’ actual needs in L2 writing at the revision stage. The data also present learners’ revision behaviours, revision processes and decisions, providing teachers who are interested in employing DDL in writing classrooms with a practical view.

### Coding and thematic analysis

Learners’ responses collected from the stimulated recall interviews were coded using thematic analysis, capturing participants’ voices and experiences in using the BAWE corpus. This could address a gap in the literature, where the presentation of data and the reporting of learners’ experiences with corpora have often been inadequate and lack in depth. Data that fulfil more aspects were revealed in this study through stimulated-recall interview responses, enabling a deeper exploration of how learners interact with corpora during the revision processes in L2 writing. The interview data consists of a total of 7.4 hours of recordings. They were first transcribed into Chinese transcripts, to around 102,500 Chinese characters. The interview data was then carefully selected based on the research questions for analysis. Afterwards, the analysed data, which were in Chinese, were translated into English for presenting the results.

Following translation, inductive coding was applied by highlighting initial codes and making a second and/or third cycle of higher-level categorised codes, as suggested by Gioia et al. [55] and Saldaña [56]. By highlighting words and phrases in the transcripts with a code, a “summative, salient, essence-capturing and/or evocative attribute” (see [56], p.3) is made visible to readers and researchers. This ensures transparency and deep immersion, allowing researchers to acquire thorough and comprehensive insights through revisiting related aspects of the data from participants’ individual views [57].

In the initial coding, affective methods were used to highlight evaluative words and phrases (e.g., *complex*; *time-consuming*) in participants’ responses. These methods were applied to capture learners’ perceptions, attitudes, and experiences of the corpus-based essay revision. Other affective coding methods, such as versus coding (e.g., *dictionary* vs. *corpus*), values coding (e.g., *the corpus is helpful* [attitude]; *proficiency affects understanding* [belief]) were also employed.

In the second cycle coding, distinctive codes were revisited and combined to refine initial codes and identify trends. The final coding cycle was to define themes. Content-based phrases related to the research questions were further organised as category labels with pattern coding (e.g., pattern code *allows pattern-seeking* with related codes *with expected answers* and *without expected answers*) and structural coding (e.g., structural code to deal with the efficacy of the corpus-based method: *efficacy + ; efficacy-*)*.* Another identified category of the codes was the distinct types of errors (i.e., WF, WC, C/P). The pattern codes, which are the meta codes identifying similar coded data, and high-frequency codes were used as themes.

### Results: Learner-corpus interactions and experiences

Regarding learners’ responses, twenty-three participants’ responses regarding how they interacted with the BAWE corpus and their experiences of the corpus-based error correction method were analysed. To resolve RQ2, the experiences of the use of the corpus-based error correction method was shown in terms of how the BAWE corpus could assist the learners to revise errors and gain linguistic knowledge, and from which aspects the use of corpus may hinder them revising lexical errors. The learner-corpus interactions were presented in extracts and their observations on the perceived effectiveness of the corpus-based error correction method was categorised into two major themes, that is, allowing pattern-seeking, and enhancing English development. Further three themes were identified in terms of the difficulties of using the corpus-based error correction method. These themes include the challenges in word selection during Key-Words-In-Context (KWIC) searching, the challenges in word selection when interpreting and applying the corpus data, and the unfamiliarity with the corpus use. Table 6 presents codes and theme patterns obtained from interview data.

**Table 6:** Coding and theme patterns.

Second cycle codes	Theme/ Third cycle coding
Pattern - success^a^ Pattern - unsuccess^b^ Reject inaccurate expressions Many samples	Allowing pattern seeking
5. More accurate/target-like 6. Shaping writing habits 7. Learn new expressions/vocabularies	Enhancing English development
8. What to search (for) 9. Others: through description	**Efficacy –** KWIC searching
10. No expected answers 11. Too many results/too much information	Applying results
12. How to operate (the corpus) 13. Not familiar 14. Too many functions	Unfamiliarity

^a^pattern – success =  allowing pattern seeking – with successful corrections.

^b^pattern – unsuccess =  allowing pattern seeking – without successful corrections

### Theme 1: Allowing pattern-seeking

One aim of implementing the corpus-based error resolution method is to assist learners to realise *why* something is an error and/or *what* the correct form is, and 21 interviewees achieved this purpose during the corpus-based error correction session. More than half of them explicitly reported that they found the accurate form after looking at the corpus data, as shown in the translated comments below:

I searched ‘topic’, and there came up with some sentences. Then I checked the left context of ‘topic’ and found that ‘hot’ is the most frequently used adjective. (S001: ‘heated [hot] topic’ - WC).

I cannot remember clearly, but I think I have searched both ‘information’ and ‘internet’ separately, and then I found that ‘on the’ was following ‘information’. (S085: ‘information of internet [on the internet]’ – C/P)

Searched ‘lack’ and I’ve got the result, no ‘ing’. (S108: ‘lacking [lack] of’ - WF)

Like the comments presented above, 17 participants in the stimulated recall interviews reflected on how the corpus data helped them to find the accurate forms of certain errors. The learners noticed the errors and proved their understanding of the accurate form of the language feature through making successful corrections. Despite a few participants in the whole study making unsuccessful corrections with the corpus-based method, six interviewees mentioned how the process of the corpus-based revision helped them realise *why* the coded item was inappropriate in the context, as exemplified below:

I used Thesaurus for this issue. I searched ‘virtue’ and had a look at its synonyms. From the results I realised that it might not be the meaning that I wanted, so I changed back to ‘benefits’. (S114: ‘virtue [benefits] of e-learning’ - WC)

When asked how S114 had come to select the word ‘virtue’, S114 said this word was simply based on the electronic dictionary’s translation. The learner explained that they wanted to choose a less-frequently occurring English word, so they made an English translation of the Chinese word 优点, and then, using this translation, decided to change it to ‘virtue’. Initially, they did not pay attention to the precise meaning of ‘virtue’. Only after searching for its synonyms in the BAWE Corpus, did they realise the shortcomings of the Chinese-English translated texts since similarities in the meaning of Chinese words may not necessarily carry over to English. Consequently, they understood that the word ‘virtue’ was inaccurate in this context.

Moreover, although four participants reported that they did not find an expected answer directly from the corpus, the corpus results aroused their knowledge of certain language points regardless of the accuracy of the corrections. This was demonstrated in the following extracts from the interview data:

I searched ‘worldwide’ and I found the results were not useful, then I tried ‘outbreak’. The results told me that most of the uses of ‘outbreak’ were ‘outbreak of’, and I looked at some more expressions of ‘outbreak of’. Then I made corrections based on the sample sentences. (S027: ‘the epidemic was outbreak worldwide [an outbreak of epidemic hit the global]’ - WF).

I have searched it in the corpus, and there was no one adding ‘s’ here, so I think it shouldn’t be an ‘s’ (here). (S086: ‘make the way of study manifolds [manifold]’ - WC)

Initially I did not know why it was an error, so I typed ‘there is one more’ and ‘one more that’ in the search box and had a look at the sample sentences…they have put something after ‘one more’. (S086: ‘there is one more that [there is one more point that]’ – C/P)

Though S027 did not make a successful correction of the target error, they had discovered the pattern of using ‘outbreak of’ and hence made a change accordingly, which was much better than the original one. Similarly, in the second example, although S086 did not successfully revise the error according to the code, they learned that ‘make’ should be followed by noun phrase and an adjective without any forms of suffix. In the last example, S086 accurately corrected the errors. Even though they could not explicitly tell what exactly should be put after ‘one more’, they proved their understanding by adding a noun after the phrase. This phenomenon is partially in line with the findings from Ellis and Shintani [58] on uptake. As highlighted in [58], by making successful corrections, learners demonstrate their understanding of the target language, achieving an “uptake with repair” (p. 207), as exemplified in the last example of the extracts. Furthermore, the findings in this study also show that even without a successful correction, uptake could still be achieved when learners realise the language points from the corpus output and apply what they have learned properly, as demonstrated in the first two examples.

The above cases suggest that learners could improve the accuracy of English writing by noticing linguistic patterns from the corpus data in two possible ways: according to the results that they found, or by analogy with instances they found in the corpus. Therefore, by consulting the corpus, the learners realised either *why* an error occurred, or *how* to revise it, showing how the knowledge could be evoked and/or acquired with consulting the corpus. This finding echoes those of some other researchers in terms of how the use of corpora could assist learners in acquiring usage patterns [6,32,46,59]. The claim of how the corpus data allows interviewees to figure out the context of word usage is also similar to the participants’ comments in [59].

### Theme 2: Enhancing english development

Some interviewees compared their previous in-class learning activities and traditional writing referencing tools with the corpus-based method, and stated that the corpus-based method could facilitate English learning by filling the teaching gap that language teachers could not cover in the classroom. Comments related to this point are shown below:

It helps me better understand those phrases that I have not fully comprehended. Indeed, it is impossible for the teacher to include everything from every aspect in English language class, so there are some phrases or words we may use inappropriately in writing. (S095)

It can definitely help you to find a more accurate expression as compared to revising by yourself or with a dictionary. (S009)

Some also expressed their opinions on how consulting the corpus could shape their habit of direct translation in English writing practice. They said,

I can learn *authentic* (more target-like) language and more accurate expressions, rather than write in Chinglish. (S085)

We students will make a direct translation from Chinese to English, using the corpus could correct those expressions so we can learn *more target-like* expressions. That is great. (S101)

You know, we all have some language habits developed from Chinese language when using English. I think using the corpus can help us eliminate this habit, helping us to write in a *more target-like* way. (S006)

As highlighted by the participants in the interviews, there was an awareness of the writing habit of direct translation. Six participants said that they always organised their thoughts in Chinese and then self-translated based on their previous knowledge or used a bilingual dictionary before writing the ideas down. They believe the BAWE Corpus provided more target-like expressions, so they could learn such expressions, and improve their English writing accuracy, simultaneously. At the same time, this monolingual corpus to a certain extent forced the users to think and search in English, which may change the learners’ habits of writing after translating. The finding on the effect of corpus consultation on reducing the interference of the first language (L1) and revising direct translations is in keeping with that of O’Sullivan & Chambers [6]. As highlighted in [6] and as shown in the present data, the results of negative transfer of L1 are more likely to be WC errors, which may also contribute to the great number of WC errors.

Furthermore, corpora can also be useful tools that enable learners to test their language hypotheses and thereby enhance the quality of their written output. Many researchers in China have encouraged learners to use less-common vocabulary in their writings [60–62]; the classroom teachers of the participants in this study tended to do the same, which may have contributed to the increased number of WC errors in their written drafts. The case of selecting the word ‘virtue’ shown in the previous theme is a typical demonstration of how Chinese English learners decide to select a less frequently used or so-called more ‘advanced’ word in English essays. In this study, many participants had chosen to change high-frequency words to low-frequency words in the second revision session (that is, to revise the drafts with dictionaries and learning materials), even though the high-frequency words were uncoded. When asked for the reasons, ten interviewees aimed to impress the raters, while the other three did so as they wanted to try using advanced vocabulary/collocations, or to write with a higher standard. The second writing session could thus be regarded as an opportunity for learners to deploy diversified vocabulary and test the appropriateness of unfamiliar language items. The third writing session (i.e., the corpus-based error correction session) could assist the learners to complete the linguistic hypotheses, improving accuracy through figuring out why the word is inappropriate and what the alternatives are.

### Theme 3: Challenges in word selection - KWIC searching

When revising an error using the corpus, the participants were required to decide which terms they needed to type into the search box, as they were not supposed to simply copy the coded expressions into the search box for all circumstances. Because of the lack of linguistic knowledge and insufficient workshop training, many participants reported that they did not know what to enter, as exemplified in these comments:

Another problem is that I do not know what to search, I have no idea how to search, so I do not know how to make the correction. (S095)

I searched ‘lecture’ and its plural form is indeed ‘lectures’, so I had no idea what else to enter in the search box. (S027: ‘from other famous lectures and schools’ - WC)

Here, after asking participant S027 the Chinese meaning that they wanted to write for the sentence, it was expected that the participant would revise ‘lectures’ to ‘lecturers’. Therefore, in this circumstance, they needed to modify the search item. However, because of insufficient training time and the unfamiliarity with the corpus, the participant failed to correct this error.

Similarly, when asked the Chinese meaning of the sentence with WC error ‘if we use heart to study’, S053 stated that they intended to mean ‘if we pay attention in class’. Obviously, the participant translated 用心word-for-word into ‘use heart’ (i.e., 用use心heart学习study). The learner knew that they should change the expression entirely but the term ‘use heart’ has no spelling or stem connection with ‘pay attention’ or other words which could express the meaning in English, so they lacked the prior knowledge to decide what to put into the search box. In this circumstance, S053 was supposed to enter the verb ‘study’ in Word Sketch because they could not think of any other suitable modifier or adverb of ‘study’. As they lacked the knowledge of the relationships within word combinations and/or within language chunks (e.g., in Word Sketch, adverb such as ‘carefully’) is categorised as the modifier of verb ‘study’ when searching for collocates of the verb ‘study’), it might have been hard for learners to determine the KWIC (e.g., key in ‘study’ in the search box of Word Sketch to find its modifier/adverb).

Many interviewees expressed their willingness to use the corpus, but they were restricted because they did not know how to search for the meanings in English. S053 mentions this in their comment below:

I have tried (to use the corpus), but I did know how to do that: I wanted to change this word but had no idea how to search. (S053: ‘we don’t have teacher look at us’ - WC)

Here, S053 was expressing the idea that teachers are not able to monitor learners in online classes. They were not able to find the target expression from the monolingual corpus relying on their own knowledge. The issue of KWIC selection has also been discussed by O’Sullivan & Chambers [6], Gilmore [15], and Mueller & Jacobson [63], as their participants also had difficulty in figuring out the KWIC for corpus query, and hence found no expected answers. Aligned with those findings, the present data suggests that the major reason for the difficulty in formulating a search query might be learners’ proficiency, but more precise reasons such as the lack of knowledge on language structure or corresponding word meanings should be considered.

### Theme 4: Challenges in interpreting and applying the corpus data

Another challenge shared by learners during the interview is the difficulty in selecting the most suitable expression from a host of corpus results. Due to the large amount of information generated by the corpus, twelve interviewees commented that they did not know which expression of the corpus output was more appropriate in their context. Examples are presented in the following extracts:

I used Context (searching) to search (for results), I wanted to find its related collocations, but there were too many results, and I did not know… I really want to use the corpus, but…you know, it’s too hard to decide. (S029).

It is wrong, I know, but I cannot find what I want from the results. I did not know which one was the right word (in Synonyms). (S114).

I did not know how to use it…there was *too much information*. For one who has lower English proficiency, it is difficult for them to choose a suitable one. (S043)

Clearly, it was hard for learners who could not analyse the corpus output to find a suitable correction. This issue is associated with a learner’s non-verbal intelligence, for instance, their analytical skills in regard to their ability to filter and select key information, and verbal intelligence such as linguistic knowledge. This aligns with previous findings, in which interpreting the corpus data is challenging for the learners due to time constraints [23,24], the amount of the corpus output [6,23], the lack of ability [24], and language proficiency [24,59,63]. Evidence regarding the effect of language proficiency has been explicitly revealed from the interview data.

When the participant was asked to recall the behaviour of making no correction when revising the phrase ‘on the one hand…on the other hand’ that was used in discussing two disadvantages of e-learning, S010 recalled that:

At that time, I searched them together, so the corpus showed me ‘Nothing Found’. But now, when I type one of them, the result appears.

Then, the participant was encouraged to provide more detail on what they found from the corpus results.

I entered ‘on the one hand’, and there is no ‘on the other hand’ followed after that expression, so I shouldn’t use ‘on the other hand’.

**Fig 4:**
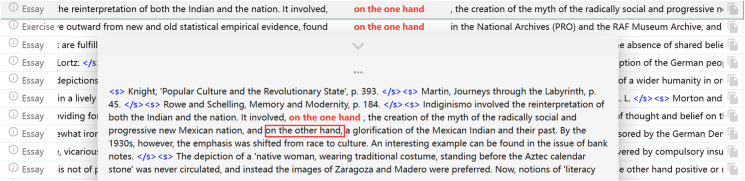
The Context of Using ‘on the one hand… on the other hand’ in the BAWE Corpus.

It is clear that this participant misinterpreted the corpus data by indicating that ‘on the one hand…on the other hand’ should not be used together. Following the conversation, the participant was instructed to look at the wider context of using this phrase from the concordance output, as shown in Fig 4. By guiding them to pay attention to the meaning of the expressions following the target phrase, they realised the linguistic pattern without being told explicitly that the phrase should be used together to compare two different or opposite facts.

The example reveals not only the role that linguistic-related factors such as the knowledge of syntactic structures play during the corpus-based revision processes, but also the value of scaffolding for lower-intermediate learners. By revealing the importance of scaffolding qualitatively, the finding echoes some previous literature which points out the need of scaffolding [25,29,33]. In particular, Chang and Sun [33] reported better concordance-assisted proofreading outcomes with written scaffolding prompts by using quantitative evidence.

### Theme 5: Unfamiliarity

This theme consists of the lack of sufficient training and hands-on instruction, the time-consuming nature of consulting the corpus, as well as the overwhelmed functions and complex interface of the corpus tool. This was reflected in most interviewees having negative experiences of using the corpus. The participants encountered technical difficulty because they had no previous experience of using the corpus and were unfamiliar with operating corpora (see also in [24]). This results in an increase in the time required for the learners to think of solutions and make corrections when searching for target expressions [6]. Consequently, the participants advocated for detailed corpus instruction and training, which aligns with what has been suggested by other researchers (e.g., [21,59]). Interestingly, the current research data does not suggest that the ability to identify suitable functions and filter information in the corpus is related to English proficiency. Some participants with low entrance scores of English (self-declared during the interview) and low self-evaluated proficiency showed themselves capable of picking up key information and making targeted corrections by consulting the corpus.

### Relationships between themes and learners’ performance

**Fig 5:**
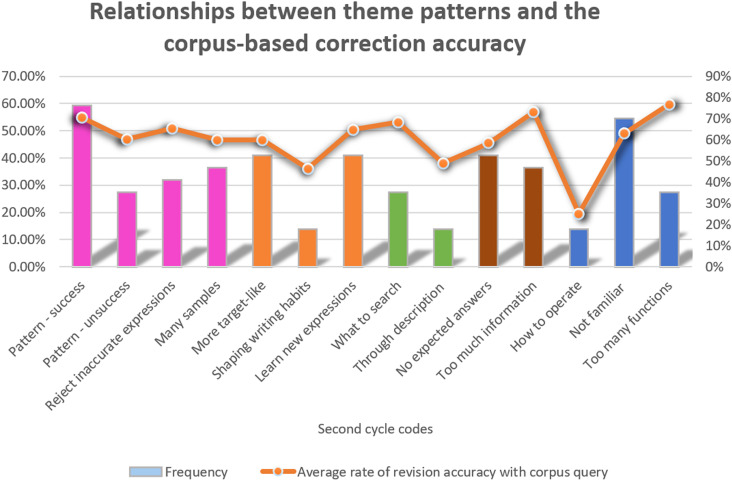
Relationships between theme frequency and the rate of corpus-based successful Corrections.

The relationships between themes frequency and the rate of successful corrections with corpus consultation are demonstrated in Fig 5. Among the interview participants, 59.1% reported that they could integrate language patterns through the corpus, which led to a high corpus-based revision accuracy rate of 70%. 27.3% of interviewees reflected that although they could not correct certain errors accurately, they could still integrate language patterns through corpus query, overall achieving an average accuracy rate of 60%. This indicates that the ability to recognize patterns using the corpus is strongly associated with successful corrections. In contrast, 54.5% of students expressed unfamiliarity with the corpus, yet their revision accuracy was relatively high (63%), suggesting that the corpus training helped them develop effective corpus query skills even if they did not feel complete familiar with the corpus.

However, those who reported not knowing how to operate the corpus faced more challenges in making accurate corrections, with the lowest rate of successful correction of 13.6%, indicating that a lack of corpus query skills significantly hampers revision success. Conversely, those who mentioned the theme of ‘too many functions’ had the highest overall correction accuracy rate of 77%, suggesting that despite the complexity, learners could effectively filter information and interpret corpus results to achieve accurate corrections.

## Discussion

With the aim of filling the gap on reporting the efficacy of the corpus-based error correction method from learners’ side, this study takes a closer look at learners’ revision process. The following sections will also discuss some key aspects of implementing the corpus-based revision method in non-English major writing classrooms in China. Some interesting findings emerge on the effectiveness of the corpus use as well as the challenges it brings to the learners during the revision processes.

### Effectiveness in correcting errors with corpora

Regarding the impact of the corpus-based error correction method on the three types of lexical errors (RQ1), the evidence suggests that it was more effectively used in revising WF errors. The present finding shows a clear tendency for the learners to address all three types of lexical errors through corpora, but clearly, the learners were more likely to resolve WF issues successfully. The quantitative result slightly diverges from those of some previous studies, as literature shows that the use of corpora can effectively help learners resolve not only WF errors, but also WC and C/P errors [22,25,31,54]. The differences may be due to learner types, the use of corpus tools, for example, the implementation of bilingual corpus query interface, or the length of training. However, according to the rates of successful correction (See Table 4), it is inconclusive to assert that the use of corpus is not effective in revising WC and C/P errors. Furthermore, the rates of successful correction for WF errors exceeded that of the other two types, regardless of reference tools (see Table 4). This may due to the nature of the errors. Making a WF error means that learners could convey their meanings semantically but fail to fit the word into the sentence syntactically [64]. In contrast, making a WC error indicates difficulty in achieving the correct semantic meaning, as learners should first select the word that fits semantically before addressing its syntactic appropriateness. Therefore, resolving WC errors requires greater cognitive effort and additional linguistic knowledge, resulting in lower accuracy rates. The production of C/P errors, similarly, suggests challenges in applying appropriate usage patterns while the learners are semantically accurate, thus demanding more effort than correcting WF errors, but less effort than correcting WC errors.

Reasons for the less favourable outcomes should be identified. Considering the nature of the corpus consultation, the less positive rates of successful correction may be due to the difficulty of interpreting the corpus data, the length of training, and the required cognitive capacity. Corpus consultation requires adequate consultation literacy and training [21], thus if learners’ corpus consulting skills and analysing skills are not be adequately developed, their choices might be affected. In addition, resolving errors of WC and C/P through corpora might occupy greater cognitive load and require higher linguistic ability. When consulting the corpus results, word form results, which are presented based on KWIC frequency, are much more clearly shown than WC and C/P results, and learners only need to determine the POS of each form and then make a correction accordingly. However, when revising WC and C/P issues, learners not only need to choose appropriate expressions based on their meanings from the corpus results which are presented in a random order, but also should pay attention to the context of using the expressions. As more processes are involved, more cognitive abilities are required.

### Learners’ decision on corpus use

The results show that learners were generally willing to use the corpus for revising lexical errors, especially C/P errors. These findings align with previous studies focusing on the corpus-based error correction method (e.g., [22,23,26]), and the present study adds to the literature by showing that the learners were willing to but less likely to successfully revise WC errors through corpora. Observing learners’ decision on corpus use during self-directed revision process offers insights into the long-term uptake of corpus use among students. As found in [65], a low uptake may be due to specific challenges faced by students. Investigating the challenges faced by learners could potentially increase the rate of uptake. In addition, as corpus consultation requires significant cognitive processing [6,15], the relationship between learners’ cognitive ability and their choice of revision method on various error types (i.e., revising WF and WC errors) can be explored in future studies.

### Overall positive perceptions towards corpus consultation

Regarding learners’ comments on the use of the corpus-based error correction method (RQ2), the stimulated recall interview responses reflect generally positive experiences of consulting the BAWE corpus. While the quantitative results indicate the effectiveness of using corpora in revising lexical issues especially on WF errors, helping learners realise *what* is the accurate form, the qualitative results further reveal its effectiveness in assisting learners to find *why* the coded item is inappropriate. The corpus query process thus enhances language learning by 1) allowing pattern detecting, which is in keeping with previous literature [6,32,46,59], 2) arousing learners’ linguistic knowledge, and 3) completing hypothesis testing

First, through pattern seeking, learners realised the difference between their original expression and the target one and thus made a successful correction, which is the evidence of language learning, as demonstrated by Ellis and Shintani [58] on learners’ uptake during the revision process. The present data also show the possibility of learners misinterpreting the linguistic patterns. Therefore, the importance of teacher or peer scaffolding should be considered when implementing corpora in writing classrooms [25,33,65]. Second, the results also suggest that even without successfully revising the errors, learners are still able to detect usage patterns from corpora results (e.g., the case of revising ‘outbreak’), or understand *why* the coded expressions were problematic (e.g., the case of revising ‘manifolds’) through arousing their acquired linguistic knowledge. Some learners were also able to find out the *why* through corpora data by comparing the context of usage of the target expressions in the corpus with their original understanding. Third, the corpus-based revision method allows the learners to undertake hypothesis testing on less frequent expressions without teacher’s feedback, which can be an effective way to learn new expression and test its usage (e.g., testing the appropriateness of using ‘virtue’). It was common among the participants in this study to try to replace some high-frequency vocabulary with lower-frequency words, and verify their understanding during the corpus-based correction session. The purpose of using the lower-frequency words was to get a higher mark, to improve writing quality, or to learn new expressions. Therefore, language teachers could consider encouraging learners to complete hypothesis-testing with the use of corpora, which not only expands learners’ vocabulary, but also trains their ability to analyse and interpret the corpus data.

### Addressing difficulties encountered by learners when consulting corpora

The main difficulties that learners encountered during the corpus-based revision process include formulating the corpus query, as noted by many researchers (e.g., [6,15,63,65,66]), interpreting and applying the corpus data (e.g., [6,15,23,42,66]), and unfamiliarity with the interface and functions [63]. In this study, the lack of understanding of ‘how to operate (the corpus)’ was closely associated with a relatively low revision accuracy. This may indicate that the difficulty in operating the corpus is a significant challenge that classroom teachers need to address, as it might negatively affect students’ correction performance. Possible reasons might be the lack of training or sufficient scaffolding. The challenge may stem from insufficient training or scaffolding before and during the revision session.

Therefore, to address these challenges, teachers could offer more training sessions with detailed illustrations, as recommended in [21] (also see [67]) and incorporate hands-on activities, as demonstrated in [68] and [69]. The issue of operating corpora may also link to unfamiliarity with the interface, and a lack of corpus query skills, which can be mitigated by providing electronic hyperlinks on useful corpus results (see [70]). Furthermore, it is suggested to develop more learner-friendly corpus query platforms to learners (e.g., allowing learners to search for the target expressions based on the frequency of hits, see [71]), which could reduce learners’ cognitive load and make corpus consultation more accessible for learners with inadequate corpus consultation skills.

## Conclusion

The current study explored the processes of learner-corpus interactions, revealing how corpora can assist learners in L2 writing development. The results conformed the effectiveness of corpus consultation in improving learners’ revision accuracy. The article also revealed how learners recognised the benefits of corpora in integrating language patterns, identifying the reasons behind errors, testing hypothesis on new language items, and searching for alternative expressions. These practices help them prevent direct translations, and improve overall writing quality.

In this regard, various teaching approaches can be adopted in L2 writing classrooms to guide learners of different proficiency levels in exploring the diverse functions of corpora, fostering both learning autonomy and motivation. Additionally, the challenges reported by participants echoed concerns raised in previous literature (e.g., [6,15,23,42,65,66]). We hope that based on the difficulties faced by students as highlighted in this paper, more user-friendly corpus software can be developed and utilised in foreign language teaching activities. Overall, the study sheds light on how corpora can be effectively integrated into language classrooms, promoting the affordances and the wide-spread adoption of corpus-based approaches in L2 writing classrooms.

There are several limitations to this study. Firstly, the answers to the efficacy of the corpus-based approach from a mixed-method perspective are not absolute and not generalisable to a wider context due to the limited sample size and research sites. Secondly, a more detailed categorisation of error types can be established. For example, distinguishing word choice problem and phrase choice problem, as well as differentiating various error types under C/P error category. Third, more detailed data could be collected to evaluate the learners’ interaction with corpora. For example, eye-tracking technology and key-stroke logging can be employed to gather richer data. In addition, as pointed out before, the relationship between learners’ cognitive ability and their choice of correction method is also suggested to be explored in future studies.
